# Single-slice ct body-weight prediction with image and metadata fusion: a multi-model comparison

**DOI:** 10.1007/s12194-026-01122-1

**Published:** 2026-08-19

**Authors:** Daniel Carrion, M. K. Badawy

**Affiliations:** 1 Radiology, Monash Health, Clayton, VIC 3168 Australia; 2 Department of Medical Imaging and Radiation Sciences, Monash University, Clayton, VIC 3800 Australia

**Keywords:** Body-weight prediction, Computed tomography, Deep learning, Multi-model comparison, Retrospective data completeness

## Abstract

A measurable fraction of examinations in retrospective imaging archives lack a recorded body weight, limiting metadata completeness for tasks such as cohort auditing, weight-stratified analysis, and the assembly of machine-learning training datasets. We developed and compared three approaches for body-weight prediction from a single middle-slice CT image: a LightGBM model on 19 CT-derived tabular features (water equivalent diameter, effective diameter, body composition, scanner parameters), an ImageNet-pretrained ResNet-18 on images alone, and a ResNet-18 with late fusion of the same 19 tabular features into the 512-dimensional image embedding. Models were trained with 5-fold patient-disjoint cross-validation on a combined train+validation development pool of 30,857 examinations from 16,169 patients; the best-performing fold of each model family was selected by within-fold validation mean absolute error (MAE) and evaluated, without further tuning, on a held-out test partition of 3,479 examinations from 1,796 patients (patient-disjoint from training and validation). On the test set, the fusion ResNet-18 achieved MAE 4.06 kg (95% CI 3.90–4.23; *R²* = 0.92), with 69.5% of predictions within ± 5 kg and 93.8% within ± 10 kg. A weighted ensemble of the fusion ResNet and LightGBM produced a small further reduction in MAE to 3.99 kg (paired bootstrap *p* < .001), but the 0.07 kg difference is below practically meaningful thresholds. The simpler single-model ResNet-18 with metadata fusion may be useful for retrospective missing-weight imputation, with split-conformal 90% and 95% prediction intervals (empirical test coverage 89% and 94%) available for case-level uncertainty quantification.

**Supplementary Information:**

The online version contains supplementary material available at https://doi.org/10.1007/s12194-026-01122-1.

## Introduction

### Background and clinical context

Large retrospective CT and PET/CT archives are increasingly mined for quality audits, dose surveys, and the assembly of machine-learning training cohorts, all of which depend on complete per-examination metadata. Body weight is a frequently missing field in such archives, and it is distinct from the patient-size metrics that CT dosimetry derives from the image: size-specific dose estimation uses the water-equivalent or effective diameter, not body weight [1–3]. Where weight is recorded, it is captured prospectively, because imaging departments operate under weight-based protocols that require a calibrated-scale measurement at the time of the examination, for example, for radiopharmaceutical activity or contrast-media volume selection [4].

The problem addressed here is retrospective. In historical archives, particularly those spanning multiple information systems, years, or data-collection regimes, a measurable fraction of examinations carry no recorded weight, either through workflow gaps or because weight logging predates current standards. Recovering these values currently requires either excluding the affected examinations or large-scale manual chart review [5, 6]. An automated estimate of body weight from images already on file would instead support retrospective uses such as assembling weight-complete cohorts for research and AI training datasets, and weight-stratified retrospective analysis and auditing.

Estimating a whole-body quantity from a single slice has some prior grounding in CT body-composition work. Mourtzakis et al. [7] reported that muscle and adipose areas measured on a single slice at the third lumbar vertebra correlate with whole-body muscle (*r* = .92) and adipose tissue (*r* = .96), with cadaver-based measurements within about 3% of dissection and coefficients of variation below 2%. That work concerned tissue composition rather than weight, so it does not validate weight prediction directly, but it indicates that a representative axial slice carries information about body size relevant to whole-body quantities.

###  Related work

#### Prior CT-based weight estimation studies

The most directly relevant prior work, published in this journal by Ichikawa et al. [8], reported MAE 1.99 kg using LightGBM with 11 tabular features extracted from whole-body PET/CT examinations on a Japanese population of 3,996 patients, demonstrating the feasibility of CT-based weight estimation and establishing water equivalent diameter (WED) and effective diameter (ED) as critical predictive features. This tabular dose-metric approach represents the current state of the art for CT-based weight prediction in the clinical medical physics literature. Earlier work by Ichikawa et al. [9] using chest radiographs for weight estimation achieved MAE 2.63–3.35 kg, demonstrating progression from 2D projection imaging to 3D CT data but with performance limitations imposed by projection geometry and positioning variability. Demircioglu et al. [10] reported MAE approximately 4–5 kg using CT scout images, an approach susceptible to patient positioning errors that cross-sectional slice analysis can avoid.

Reported accuracy spans volumetric tabular-feature pipelines (best reported MAE 1.99 kg [8]) and simplified single-slice or scout-image approaches (MAE 2.63–5 kg [9, 10]). Whether a single axial slice combined with acquisition metadata retains sufficient information for accurate weight estimation and how much accuracy is lost relative to whole-volume features has not been systematically characterised.

#### Gap analysis

Despite these advances, several gaps remain. Direct comparisons between gradient boosting on engineered tabular features and end-to-end deep learning on the image itself, with both methods evaluated on the same large cohort, have not been reported for CT-based weight estimation. Whether late fusion of acquisition-metadata features into a deep convolutional embedding adds incremental value over either approach alone has not been systematically characterized in this domain. Reporting of test-set performance against patient-level confidence intervals, rather than examination-level intervals that underestimate uncertainty in multi-slice-per-patient cohorts, is also uncommon in prior CT body-weight work. This study addresses these gaps using LightGBM [11] with Gain-based feature importance, ResNet-18 [12] with ImageNet transfer learning [13] and late metadata fusion [14], and weighted ensemble averaging [15, 16].

###  Study objectives

This study addresses the identified gaps through systematic comparison of gradient boosting, deep learning with and without metadata fusion, and ensemble approaches for body-weight prediction from a single middle-slice CT image. The aim is to characterise what a single slice combined with acquisition metadata can achieve, not to match the accuracy of volumetric approaches that use the full CT volume. The objectives are:

1. To develop and compare feature-engineered gradient boosting (LightGBM), images-only deep learning (ResNet-18), and a fusion variant integrating 19 CT-derived tabular features into the ResNet-18 image embedding via late concatenation, with feature-importance analysis of the gradient-boosting model.

2. To evaluate an optimized weighted ensemble of the fusion ResNet and LightGBM, and to test whether its improvement over the single-model ResNet-18 CT Fusion is statistically significant by patient-level paired bootstrap.

3. To report all primary performance numbers on a **held-out test partition** that was patient-disjoint from training and validation, with patient-level bootstrap confidence intervals and out-of-fold split-conformal prediction intervals for per-case uncertainty quantification.

##  Materials and methods

### Data and ethical approval

#### Dataset characteristics

The dataset comprised 34,336 middle-slice CT images extracted from 17,965 unique patients (average 1.9 examinations per patient) who underwent whole-body PET/CT between 2015 and 2025. This cohort was selected because F-18 FDG dosing and SUV normalisation both require a contemporaneous body weight, so every patient was weighed on a calibrated scale before injection as part of standard clinical protocols; all included patients therefore had measured and verified reference weights. Demographic and acquisition characteristics are summarized in Table 1. Images were acquired across 131 unique scan protocols, with tube voltage ranging from 70 to 150 kVp (majority 120 or 140 kVp) and reconstructed slice thickness 1.5 to 10.0 mm (mean 4.0 mm). Automatic tube current modulation was employed across most acquisitions.

#### Study design and ethical compliance

This study was conducted retrospectively on de-identified imaging data obtained for clinical purposes. The Monash Health Human Research Ethics Committee determined that the study was exempt from formal review as a retrospective quality improvement project, consistent with the *Ethical Considerations in Quality Assurance and Evaluation Activities* guideline (National Health and Medical Research Council, 2014). The study adheres to the principles of the Declaration of Helsinki (1964, and subsequent amendments). Inclusion required a contemporaneous body weight measurement; exclusions were missing weight records, incomplete CT metrics, severe motion artifacts, and incomplete acquisition protocols. All data were de-identified by removing protected health information from DICOM headers prior to analysis. The reference standard was a calibrated-scale physical measurement recorded as part of routine radiopharmaceutical dosing, requiring no subjective annotation; inter- or intra-rater variability does not apply.

The source PET/CT archive contained 37,178 CT acquisitions from 20,291 patients; after filtering for complete weight, CT metric, and demographic data, 34,336 slices from 17,965 patients were retained. The total sample is approximately fourfold larger than the largest prior CT-based weight estimation study (Ichikawa et al. [8]: 3,996 patients) and substantially exceeds other published cohorts [9, 10], providing statistical power for stable regression estimates.

#### Data partitioning

The 17,965 patients (34,336 slices) were partitioned into three sets at the **patient level** using a fixed random seed (scikit-learn v1.0.2 train_test_split, seed = 42), in two stages: train vs. (validation+test) at ratio 80:20, then validation vs. test within the held-out 20% at ratio 50:50. All slices belonging to a single patient were assigned to the same partition, ensuring zero patient overlap across pairs. The resulting partitions were 14,373 patients (27,329 slices) train, 1,796 patients (3,528 slices) validation, and 1,796 patients (3,479 slices) test, verified by direct database query (0 patient overlap across all three pair-wise comparisons). Distributional balance across partitions for weight, age, sex, and tube voltage was achieved by the law of large numbers given the cohort size rather than by explicit stratification on these variables; Table 1 documents the resulting distributions and confirms they are comparable across partitions.

The fixed validation partition carries the same fold labels as the training pool and is therefore not independent of the cross-validation models (§ 2.4.1); it was used only during development (ensemble mixing-weight selection) and is not reported as an evaluation set. The test partition was held out from all training, model selection, and weight tuning, and constitutes the primary, unbiased performance evaluation reported in Sect.  3.

### 2Data preprocessing and feature extraction

#### DICOM processing and intensity normalization

Raw DICOM images underwent standardized preprocessing to ensure consistency across multi-vendor acquisitions. Pixel intensity values were converted from stored values to Hounsfield Units (HU) using the rescale slope and intercept parameters embedded in DICOM headers. Intensity windowing was applied using soft-tissue window settings (window center: 40 HU, window width: 400 HU) to enhance contrast for body composition analysis. This windowing approach emphasized adipose tissue (-190 to -30 HU), muscle (30 to 100 HU), and visceral structures while suppressing bone and air regions.

####  Middle-slice selection methodology

A consistent positional reference was established by selecting the middle axial slice from each CT examination, defined as the median slice index along the superior-inferior axis. The geometric middle was chosen deliberately because it requires no anatomical landmark detection and is therefore reproducible across heterogeneous scan types without an additional model in the pipeline. This is a practical consideration for batch processing of large retrospective archives. In this cohort the great majority of examinations are standardized whole-body PET/CT acquisitions (93.9% of slices; craniocaudal extent median 1,916 mm), for which the geometric center reliably approximates the abdomen/pelvis level and provides representative cross-sectional body composition. We acknowledge that, for limited-coverage acquisitions, the middle slice falls at a more variable anatomical level; the empirical effect of this variability on accuracy is quantified by scan-coverage subgroup analysis (§ 3.11, Table S3), and an anatomically standardized alternative (e.g., L3-vertebra–based selection) is discussed as future work in § 4.7. The geometric approach is also consistent with prior single-slice morphometric approaches.

#### CT Metrics extraction and feature engineering

Quantitative CT metrics were extracted from the selected middle slice using custom Python-based image analysis algorithms implemented with NumPy (v1.21.0), SciPy (v1.7.1), and scikit-image (v0.20) libraries. The water equivalent diameter (WED) was calculated as a standardized measure of patient size, accounting for the differential attenuation of various tissues relative to water, following AAPM Task Group 220 guidelines. WED accounts for tissue composition variations in patient-specific dose estimation through the relationship:

$$\:\mathrm{WED}=\mathrm{ED}\times\:\sqrt{\frac{1000+\mathrm{mean\_HU}}{1000}}$$

where ED represents effective diameter [2].

The effective diameter (ED) was computed as a geometric measure of patient cross-sectional size, calculated from the total area of tissue-containing pixels above a threshold of -500 HU (excluding air). Body composition metrics were derived through HU-based tissue segmentation employing validated Hounsfield unit thresholds: muscle tissue (30 to 100 HU), adipose tissue (− 190 to − 30 HU). Scanner and acquisition parameters extracted from DICOM metadata included tube voltage (kVp), tube current-time product (mAs), slice thickness, pixel spacing, table height, source-to-patient distance, and generator power. Generator power represents the DICOM tag (0018,1170), recording the X-ray generator’s rated power output in kilowatts.

The final feature set comprised 19 directly measured and computed variables (polynomial and interaction terms did not improve cross-validation performance and were excluded), spanning morphometric measurements (WED, ED, cross-sectional area, body pixel count), body composition metrics (total tissue, subcutaneous fat, visceral fat, liver, and muscle areas), and scanner acquisition parameters (kVp, mAs, tube current, exposure time, CTDIvol, SSDE, generator power, table height, source-to-patient distance, pixel spacing). Missing metadata values (absent kVp, mAs, or generator power fields in DICOM headers) were imputed with population-level medians from the training set.

#### Image standardization for deep learning

CT images for ResNet-18 input were resized to 224 × 224 pixels (bilinear interpolation) to match the pre-trained network’s input dimensions. The identical resize was applied to every partition, including the held-out test set, so that training-time and evaluation-time preprocessing are matched. Intensity values were min-max scaled to [0, 1] after windowing, and the grayscale channel was replicated to three channels to match the pre-trained network input. No augmentation was applied; preliminary experiments with random horizontal flips and ± 10° rotations showed no improvement in validation MAE (differences < 0.05 kg), consistent with the standardized orientation of axial CT slices.

### Proposed methods

#### LightGBM gradient boosting framework

The gradient boosting model was implemented using the LightGBM framework, which employs histogram-based gradient boosting decision trees optimized for regression tasks [11].

Given input features $\:\mathbf{x}\in\:{\mathbb{R}}^{19}$, the model output is the sum of * T* sequentially trained decision trees:

1 $$\:\widehat{y}=\sum\:_{i=1}^{T}{f}_{i}\left(\mathbf{x}\right)$$

The model was trained for up to 5,000 boosting iterations with learning rate 0.01, minimizing mean squared error. Hyperparameters were tuned by Bayesian optimization with five-fold cross-validation on the training set (max depth 8, min data in leaf 20, feature fraction 0.8, bagging fraction 0.8 every 5 iterations, L1/L2 regularization 0.1), with early stopping at 100 rounds on validation MAE. Training was performed on a 16-core CPU in 74.8 min total.

#### ResNet-18 convolutional neural network variants

Two ResNet-18 model variants were developed to evaluate the contribution of metadata fusion. ResNet-18 was chosen rather than deeper residual variants (ResNet-34, ResNet-50) for three reasons: (i) the cohort size (~ 34,000 slices) is well-matched to ResNet-18’s ~ 11 M parameters and does not motivate deeper networks that would increase overfitting risk without performance benefit; (ii) inference latency is sub-millisecond per slice on an NVIDIA Tesla P40, which supports the retrospective batch-processing use case; and (iii) ResNet-18 has been used in prior single-slice CT regression tasks with cohorts of similar scale [12]. Both variants were initialized with ImageNet pre-trained weights to leverage transfer learning, with the original classification head removed and the final 512-dimensional feature vector from the global average pooling layer retained as the learned image representation.

ResNet-18 Images Only: A baseline variant processing CT images exclusively. The 512-dimensional image feature vector was passed through a fully connected regression head (512 → 256 → 64 → 1) with ReLU activations and dropout (*p* = .3) after the first two layers.

ResNet-18 CT Fusion: A variant integrating the same 19-dimensional tabular feature vector used by LightGBM (morphometric measurements, body composition metrics, and scanner parameters) with image features through late concatenation.

2 $$\:{\mathbf{f}}_{\mathrm{fused}}=[{\mathbf{f}}_{\mathrm{image}};{\mathbf{f}}_{\mathrm{meta}}]$$

producing a 531-dimensional representation passed through a regression head (531 → 256 → 64 → 1) with the same activations and dropout. The final output is a single continuous value representing predicted patient weight in kilograms. This dual-variant design enables direct assessment of the metadata fusion contribution while holding the base architecture constant.

####  Training procedure and optimization

ResNet variants were trained with Adam [17] (initial learning rate 0.0001, halved on validation-loss plateau over 5 epochs), batch size 32, weight decay $\:1\times\:{10}^{-4}$, and MSE loss for up to 200 epochs with early stopping on validation MAE (patience 15); the final model was the epoch with lowest validation MAE. Training used an NVIDIA Tesla P40 GPU with FP16 mixed precision. Hyperparameters were selected from a brief manual sweep on fold 0 and held fixed across folds and both ResNet variants to keep comparisons fair, the focus being model comparison rather than absolute performance optimization.

#### Ensemble weighted averaging

An ensemble was constructed by combining each ResNet-18 variant with LightGBM through weighted linear averaging:

3 $$\:{\widehat{y}}_{\mathrm{ensemble}}={w}_{\mathrm{ResNet}}\cdot\:{\widehat{y}}_{\mathrm{ResNet}}+(1-{w}_{\mathrm{ResNet}})\cdot\:{\widehat{y}}_{\mathrm{LightGBM}}\:$$

where $\:{w}_{\mathrm{ResNet}}$ is the mixing weight assigned to the ResNet variant. The mixing weight was selected on the validation partition by exhaustive grid search at 1% increments ($\:{w}_{\mathrm{ResNet}}$: 0.00 to 1.00), minimizing validation MAE. The procedure yielded $\:{w}_{\mathrm{ResNet}}=.84$ for the CT Fusion ensemble (0.84 ResNet-18 CT Fusion + 0.16 LightGBM) and $\:{w}_{\mathrm{ResNet}}=.66$ for the Images Only ensemble (0.66 ResNet-18 Images Only + 0.34 LightGBM). These weights were frozen after validation-set selection and were not retuned on the test partition; the test-set ensemble performance reported in Sect.  3 is therefore an unbiased evaluation of the combined model.

###  Performance evaluation framework

####  Two-stage evaluation strategy

Model performance was evaluated in two stages, distinguishing development-time validation from final unbiased test-set evaluation:

**Stage 1: model development and selection (k-fold CV on the development pool).** The training and fixed-validation partitions were combined into a single development pool of 30,857 slices from 16,169 patients (the held-out test partition, *n* = 3,479, was excluded entirely) and divided into five patient-stratified folds, with each patient assigned to exactly one fold (~ 6,171 slices per fold). Consequently the fixed validation partition is not an independent hold-out for the resulting models; it forms part of the cross-validation pool. For each fold, the complete training pipeline (LightGBM Bayesian hyperparameter search; ResNet-18 200-epoch Adam training with early stopping on the held-out fold’s MAE) was executed. Within each model family, the fold with the lowest held-out MAE was selected as the family’s representative model: ResNet-18 CT Fusion fold 3 (within-fold validation MAE 3.81 kg), ResNet-18 Images Only fold 0 (4.28 kg), and LightGBM fold 3 (5.25 kg). Pooling every fold’s held-out predictions yields *n* = 30,857 out-of-fold predictions per base model; the optimized ensemble, which requires aligned out-of-fold predictions from both component models, is available for *n* = 30,497 of these and underlies the subgroup and failure analyses (§ 3.9–3.10). The deployed CT Fusion model’s own held-out fold provides the *n* = 6,171 out-of-fold residuals used to calibrate prediction intervals (§ 2.5). Because the test partition was excluded from this pool and from all model selection, hyperparameter tuning, and ensemble-weight optimization, it provides the single unbiased evaluation, described next.

Stage 2: final evaluation on the held-out test (*n* = 3,**479 slices from 1**,**796 patients).** The three k-fold-best models were then evaluated, without further tuning, on the held-out test partition described in § 2.1.3. Ensemble mixing weights $\:{w}_{\mathrm{ResNet}}$ (§ 2.3.4) were selected on the validation partition and frozen; because that partition is not independent of the fold-best models, the unbiased assessment of those frozen weights is their performance on the held-out test partition, where they were not retuned. As an alternative to the fold-best selection rule, retraining each ResNet variant on the full training partition after selection yields comparable held-out test performance (Table S4), confirming the selection rule does not introduce optimistic bias. All headline numerical results reported in Sect.  3 are derived from this stage.

#### Performance metrics

Test-set performance for each of the five models (three base + two ensembles) was characterized by absolute, relative, and tolerance-based metrics:

• **Absolute error**: mean absolute error (MAE), root mean squared error (RMSE), coefficient of determination (*R²*), mean bias (predicted − actual). MAE was the primary endpoint.

• **Relative error**: median absolute percentage error (MAPE) with interquartile range; high-percentile absolute errors (P90, P95) to characterize tail behavior.

• **Accuracy thresholds**: fraction of predictions within ± 5 kg, ± 10 kg, ± 5%, and ± 10% of the true weight. The 10% threshold corresponds to a commonly used weight-variance tolerance.

• **Bland–Altman analysis**: mean bias and 95% limits of agreement (LoA).

• **Subgroup performance**: per-bin metrics across weight, sex, age, and tube voltage strata.

• **Feature importance**: LightGBM native Gain-based importance [11] (not SHAP) for the gradient-boosting model, providing interpretation of which CT-derived features contribute most to weight prediction.

### Statistical analysis

All statistical computations were performed in Python 3.8 with NumPy v1.21.0 and SciPy v1.7.1.

**Confidence intervals.** Bootstrap 95% CIs (10,000 resamples) were computed at the **patient level**, not the examination level: each bootstrap iteration resampled patient identifiers with replacement (with sample size equal to the number of unique patients in the partition), then aggregated all slices for the sampled patients. This procedure accounts for intra-patient correlation arising from multi-slice patient contributions and yields wider, honest CIs than examination-level resampling would.

**Paired model comparison.** The ensemble (CT Fusion + LightGBM, 84/16) was compared with the standalone ResNet-18 CT Fusion using patient-level paired bootstrapping (10,000 iterations): within each iteration, the same resampled set of patients was scored under both models, and the MAE difference was retained. Two-sided p-values were derived as $\:2\cdot\:\mathrm{min}\left\{P\right(\varDelta\:\le\:0),P(\varDelta\:\ge\:0\left)\right\}$.

**Prediction intervals.** Split-conformal 90% and 95% prediction intervals (PIs) for ResNet-18 CT Fusion were calibrated on the deployed model’s out-of-fold residuals from cross-validation (the fold-3 model’s predictions on its held-out fold, *n* = 6,171), which the model never saw during training. For target coverage $\:1-\alpha\:$, the interval half-width is q, the $\:\lceil\:(n+1)(1-\alpha\:)\rceil\:$-th smallest absolute calibration residual (finite-sample valid), giving the symmetric interval $\:[\widehat{y}-q,\widehat{y}+q]$. Empirical coverage was then evaluated on the held-out test partition, and a reliability diagram across nominal levels was constructed (Fig. S5). The fixed validation partition was not used for calibration because it is not independent of the fold-best models (§ 2.4.1).

**Subgroup tests.** Differences in continuous metrics across subgroups (weight, age, sex, tube voltage strata) were tested with Kruskal–Wallis where appropriate; results are reported descriptively without formal omnibus correction.

**Reproducibility.** A fixed random seed of 42 was used for all stochastic procedures (partition assignment, bootstrap resampling, ensemble grid search, neural network initialization). All training, evaluation, and statistical scripts are available on request.

All models were implemented in Python 3.8 using PyTorch v1.12.1, LightGBM v3.3.2, pydicom v2.3.0, NumPy v1.21.0, SciPy v1.7.1, scikit-learn v1.0.2, Matplotlib v3.5.1, and seaborn v0.11.2.

### Generative AI disclosure

Large-language-model assistance (Anthropic Claude) was used during manuscript preparation for stylistic editing of author-written text only. The LLM was not used to design the study, generate or alter primary data, perform statistical analyses, or create figures or tables. All scientific content was originated, verified, and approved by the human authors, who take full responsibility for the manuscript.

## Results

### Cohort characteristics

Table 1 summarizes demographic and acquisition characteristics across the three patient-disjoint partitions. Weight, age, sex, and tube voltage distributions are comparable across train, validation, and test partitions; chi-squared (sex) and Kruskal–Wallis (continuous) tests confirmed no statistically significant cross-partition differences (all *p* > .15).

**Table 1:** Demographic and clinical characteristics by data partition

Characteristic	Train (*n* = 27,329 slices, 14,373 patients)	Validation (*n* = 3,528 slices, 1,796 patients)	Test (*n* = 3,479 slices, 1,796 patients)
Male, n (%)	14,177 (51.9%)	1,856 (52.6%)	1,763 (50.7%)
Female, n (%)	13,149 (48.1%)	1,672 (47.4%)	1,716 (49.3%)
Age, mean ± SD (years)	63.3 ± 15.7	63.2 ± 15.3	63.2 ± 16.0
Weight, mean ± SD (kg)	77.0 ± 20.8	77.5 ± 19.5	77.1 ± 20.2
Weight range (kg)	5.7–238.2	10.7–185.5	7.8–200.0
Tube voltage (kVp), mean ± SD	122.5 ± 12.4	122.9 ± 11.9	122.3 ± 12.4

Sex was recorded as male or female for all slices except three in the training partition (two recorded as other, one unspecified); percentages are of the full partition

### Held-out test-set performance

**Fig. 1:**
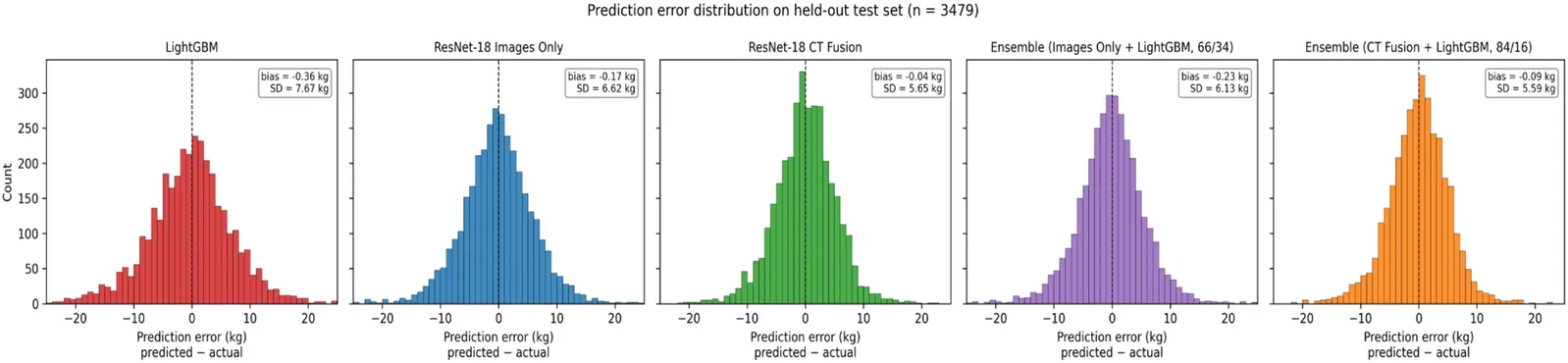
Error distribution on the held-out test set (*n* = 3,479) for each of the five models. Histograms show signed prediction error (predicted − actual) in 1 kg bins. ResNet-18 CT Fusion and the CT Fusion + LightGBM ensemble show the narrowest distributions; LightGBM shows the widest, with slightly heavier tails Error histogram by model on held-out test set

Table 2 reports performance on the held-out test partition (*n* = 3,479 slices from 1,796 patients) for all five models. Patient-level bootstrap 95% CIs were computed by resampling 1,796 unique patient identifiers with replacement (10,000 iterations), aggregating all slices for each sampled patient. ResNet-18 CT Fusion achieved the best individual-model performance (MAE 4.06 kg [95% CI 3.90–4.23], *R²* = 0.92), outperforming ResNet-18 Images Only (MAE 4.73 [4.52–4.96]) by 14.2% and LightGBM (MAE 5.61 [5.37–5.87]) by 27.6%. The weighted ensemble of ResNet-18 CT Fusion and LightGBM (84/16) produced the best overall MAE of 3.99 kg [3.83–4.16] (Table 2; Fig. 1).

**Table 2:** Held-out test-set performance for all five models. Mean absolute error (MAE) reported with patient-level bootstrap 95% confidence intervals (10,000 iterations, seed 42)

Model	MAE (kg) [95% CI]	RMSE (kg)	*R*²	Mean bias (kg)
LightGBM	5.61 [5.37–5.87]	7.68	0.856	−0.36
ResNet-18 Images Only	4.73 [4.52–4.96]	6.62	0.893	−0.17
ResNet-18 CT Fusion	4.06 [3.90–4.23]	5.65	0.922	−0.04
Ensemble (Images Only + LightGBM, 66/34)	4.33 [4.13–4.54]	6.13	0.908	−0.23
**Ensemble (CT Fusion + LightGBM**,** 84/16)**	**3.99 [3.83–4.16]**	**5.59**	**0.924**	**−0.09**

RMSE and *R²* are point estimates. Bold indicates lowest MAE

**Fig. 2:**
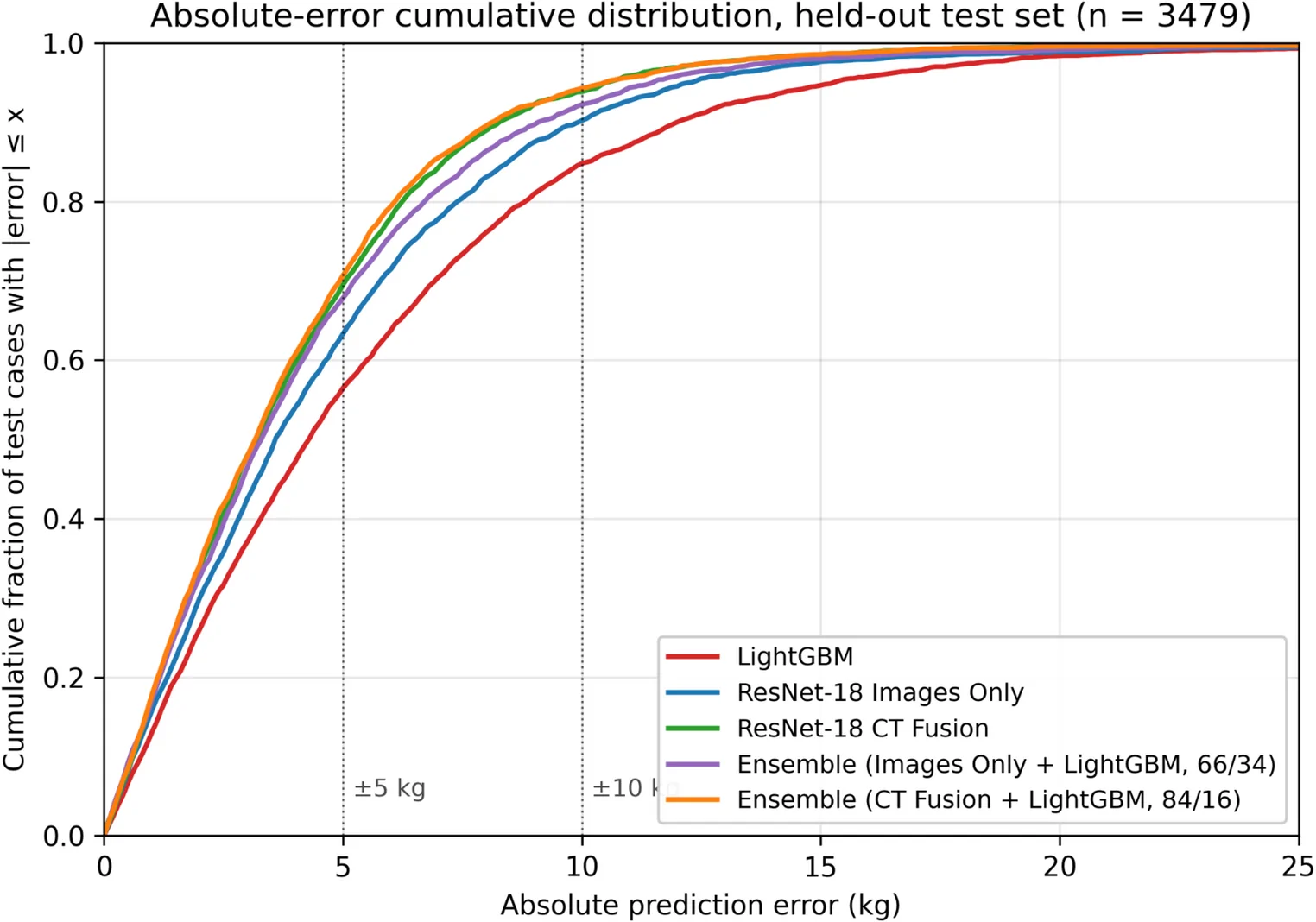
Cumulative distribution of absolute prediction error on the held-out test set. For each model, the curve indicates the fraction of test cases with |error| ≤ *x* kg. Vertical dashed lines mark the ± 5 kg and ± 10 kg thresholds. Ensembles and ResNet-18 CT Fusion cluster above ResNet-18 Images Only and LightGBM throughout Cumulative absolute-error CDF by model on held-out test set

### Accuracy and tail behaviour

Table 3 summarizes relative error and tolerance-based performance on the test set, complementing the absolute-error metrics in Table 2.

**Table 3:** Relative error, tail percentiles, and tolerance-based accuracy on the held-out test set

Model	MAPE (%) median [IQR]	P90	err	(kg)	P95	err	(kg)
LightGBM	5.80 [2.65–10.26]	12.00	15.24	56.5%	84.8%	44.5%	73.9%
ResNet-18 Images Only	4.94 [2.28–8.63]	9.92	12.25	63.3%	90.2%	50.5%	80.8%
ResNet-18 CT Fusion	4.28 [1.92–7.55]	8.27	10.49	69.5%	93.8%	56.4%	86.5%
Ensemble (Images Only + LightGBM, 66/34)	4.51 [2.02–7.96]	9.06	11.46	67.8%	92.2%	54.9%	84.7%
**Ensemble (CT Fusion + LightGBM**,** 84/16)**	**4.29 [1.85–7.33]**	**8.14**	**10.48**	**70.7%**	**94.3%**	**56.6%**	**87.0%**

MAPE is the median absolute percentage error with interquartile range; P90 and P95 are 90th and 95th percentiles of absolute error

Within the ± 10 kg tolerance, 94.3% of ensemble predictions and 93.8% of ResNet-18 CT Fusion predictions fell inside on test; on the relative ± 10% benchmark (a commonly used weight-variance tolerance) the corresponding figures were 87.0% and 86.5%. Tail behavior was comparable between the two top performers: 95% of test cases had absolute error ≤ 10.5 kg (P95).

### Paired comparison: ensemble vs. ResNet-18 CT fusion

A patient-level paired bootstrap (10,000 iterations) compared the CT Fusion + LightGBM ensemble against ResNet-18 CT Fusion on the held-out test set. The observed MAE difference (Ensemble − CT Fusion) was **− 0.073 kg** (patient-level 95% CI − 0.113 to − 0.032 kg; two-sided *p* < .001). The improvement is therefore statistically robust but corresponds to only ~ 0.1% of mean test-set body weight. Section  4 discusses the practical and deployment implications of this difference.

### Prediction intervals

Split-conformal 90% and 95% prediction intervals (PIs) for ResNet-18 CT Fusion were calibrated on the model’s genuinely out-of-fold residuals from cross-validation (*n* = 6,171); the fixed validation partition was not used for calibration because the k-fold-best models were exposed to most of it during fold training (§ 2.4.1). The 90% PI is prediction ± 8.00 kg (width 16.0 kg) with empirical held-out-test coverage of 89.0%; the 95% PI is prediction ± 9.87 kg (width 19.8 kg) with empirical test coverage of 93.8%. Both intervals achieve close to nominal coverage, in contrast to a naive calibration on the contaminated validation partition, which substantially undercovers (61.1% and 75.5%); a reliability diagram across nominal levels is provided in Fig. S5. PI widths exceed the population-level ± 5 / ±10 kg tolerance thresholds reported in Table 3, reflecting per-case rather than population-average uncertainty (Fig. 2).

### Bland–altman agreement

**Fig. 3:**
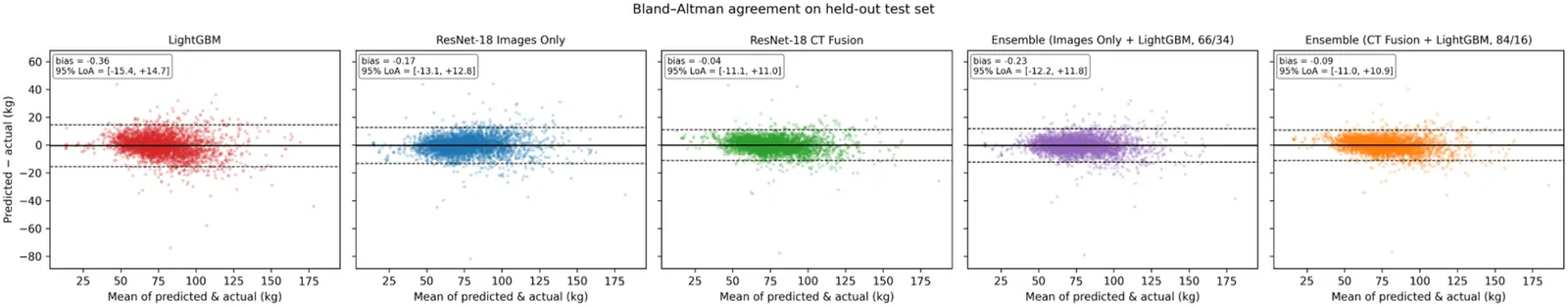
Bland–Altman plots per model on the held-out test set. The solid horizontal line in each panel marks the mean bias; the dashed lines mark the 95% limits of agreement (mean bias ± 1.96 × SD) Bland-Altman plot per model on held-out test set

Bland–Altman analysis on the held-out test set (Fig. 3) showed mean bias of − 0.09 kg for the CT Fusion + LightGBM ensemble (consistently small across models, range − 0.36 to − 0.04 kg) with 95% limits of agreement spanning approximately ± 11 kg. The narrow mean bias does not imply tight individual-case agreement; it summarizes the population-average prediction tendency, whereas the LoA characterizes individual-case variability. Section  4.2 discusses this accuracy-versus-precision distinction and its implications for population- and cohort-level use.

### Predicted vs. actual scatter

**Fig. 4:**
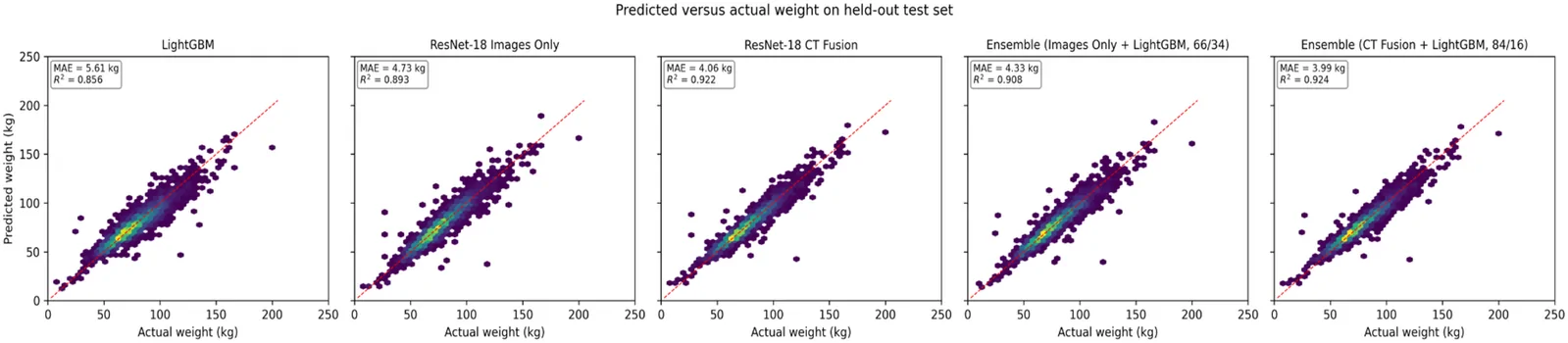
Predicted versus actual weight on the held-out test set per model. Identity line (*y* = *x*) is shown for reference. Color intensity indicates data density. Best-performing models (ResNet-18 CT Fusion and the CT Fusion + LightGBM ensemble) cluster more tightly around the identity line Predicted vs. actual weight per model on held-out test set

Scatter plots of predicted versus actual weight (Fig. 4) confirm strong linear relationships for ResNet-18 CT Fusion and the ensemble (*R²* = 0.92 and 0.92). LightGBM and ResNet-18 Images Only show greater dispersion. All models show evidence of shrinkage toward the cohort mean (~ 77 kg), manifested as slight overestimation at low weights and underestimation at high weights, a common pattern in regression-to-mean behavior discussed in Sect.  4.

### Feature importance

**Fig. 5:**
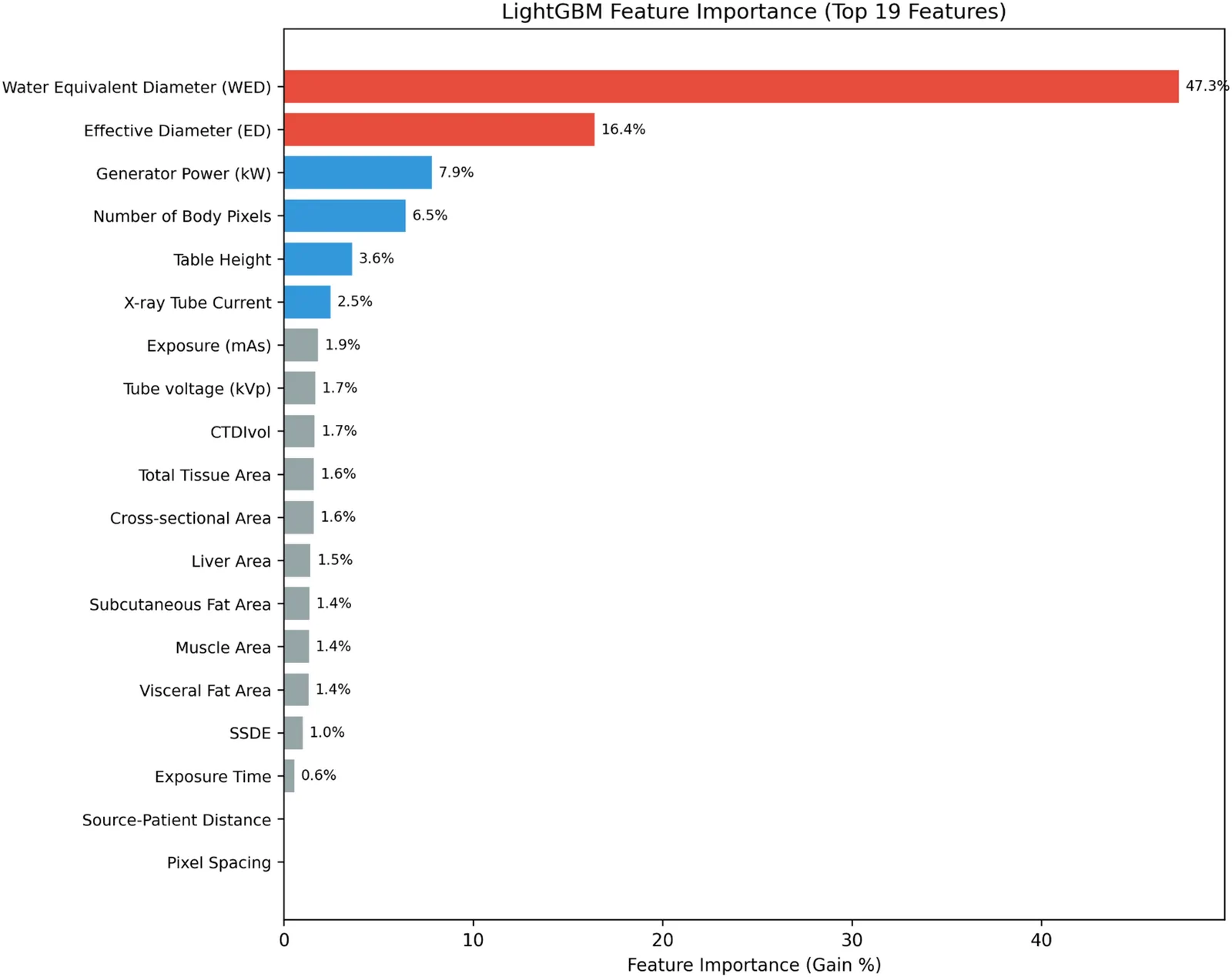
Feature importance ranking (LightGBM native Gain importance) for the 19 CT-derived tabular features. WED contributes 47.3% of total importance, followed by ED (16.4%); the remaining 17 features each contribute below 10% LightGBM feature importance (Gain)

 Figure 5 shows the feature-importance (Gain) ranking from the LightGBM model, computed as the mean across the five cross-validation folds. Water equivalent diameter (WED) dominated (47.3% of total Gain), followed by effective diameter (16.4%), generator power (7.9%), and body pixel count (6.5%). The dominance of WED and ED is consistent with their role in AAPM Task Group 220 size-specific dose estimation [2, 3], where these geometric metrics are the primary determinants of patient-size correction.

### Subgroup performance

For high-resolution subgroup analyses, the per-subgroup sample size on the held-out test partition (*n* = 3,479) becomes limited at weight extremes (e.g., < 40 kg: *n* = 35 on test). To maintain statistical power, subgroup performance is therefore reported on the 5-fold out-of-fold ensemble predictions (*n* = 30,497; § 2.4.1), where each slice was predicted by the fold model that did not see it during training (Table 4). This is methodologically distinct from but complementary to the held-out test evaluation in Tables 2 and 3.

**Table 4:** Ensemble (CT Fusion + LightGBM, 84/16) performance by weight category from the 5-fold out-of-fold ensemble predictions (*n* = 30,497)

Weight (kg)	*n*	MAE (kg)	RMSE (kg)	Within-bin *R*²	Within ± 5 kg
< 40	353	6.04	10.48	−0.173	55.5%
40–60	5,466	3.29	4.43	0.191	78.5%
60–80	12,383	3.28	4.34	0.419	78.8%
80–100	8,432	3.97	5.34	0.082	70.8%
100–120	2,824	5.26	6.94	−0.539	56.5%
> 120	1,039	8.06	14.13	0.178	44.0%

Within-bin *R²* values are reported; small or negative within-bin *R²* reflects the narrow weight variance within each bin and does not contradict the cross-cohort fit reported in Table 2

Performance was strongest in the 40–80 kg range (MAE 3.28–3.29 kg, 78.5–78.8% within ± 5 kg) and degraded substantially at extremes (< 40 kg: MAE 6.04 kg; >120 kg: MAE 8.06 kg, 44.0% within ± 5 kg). Within-bin *R²* values are small or negative for several weight categories, most notably 100–120 kg (*R²* = −0.539). These reflect that the model captures cohort-wide weight trends but cannot finely rank-order patients within a narrow ~ 20 kg window, where prediction noise is comparable to within-bin variance; this does not contradict the cross-cohort test *R²* of 0.92, and indicates the practical use case is **population-level estimation** rather than within-narrow-band ranking. Predictions for patients < 40 kg or > 100 kg should be flagged as lower-confidence and may warrant confirmatory measurement.

By sex, performance was equivalent (female MAE 3.82 kg; male MAE 3.88 kg). By age, the pediatric subgroup (< 18 years, *n* = 506) performed well (MAE 3.27 kg, *R²* = 0.967, 79.4% within ± 5 kg). Performance varied with tube voltage: examinations acquired at < 120 kVp showed lower MAE (3.15–3.28 kg) than examinations acquired at > 140 kVp (MAE 5.05 kg; Kruskal–Wallis *p* < .0001), consistent with the association between scanner-protocol selection and body habitus.

### Failure analysis

Failure analysis on the highest-error cases (|error| ≥ 10.0 kg; *n* = 1,511, ~ 5% of the 30,497 out-of-fold ensemble predictions) showed that high-error cases were skewed toward heavier patients (median weight 93.0 kg, IQR 75.0–112.0 kg, versus the development-pool mean of 77.1 kg; *p* < .0001). Error direction was predominantly underestimation (mean error − 3.9 kg among failure cases; 38.0% overestimation versus 62.0% underestimation). Sex distribution among failure cases was similar to the development cohort. These patterns are consistent with the subgroup observations in § 3.9 and motivate the case-level uncertainty flagging discussed in Sect.  4.

### Sensitivity analyses

**One examination per patient.** To confirm that the ~ 1.9 examinations per patient do not inflate the held-out result, the test partition was restricted to a single examination per patient (earliest study date; *n* = 1,796). All five models changed by ≤ 0.08 kg in MAE and showed marginally higher *R²* (ResNet-18 CT Fusion 4.06 → 4.02 kg, *R²* 0.92 → 0.93; ensemble 3.99 → 3.95 kg), confirming the reported performance is not an artifact of repeated examinations (**Table S2**).

**Scan coverage and anatomical variability.** Because the geometric middle slice can fall at different anatomical levels depending on scan coverage, test-set performance was stratified by acquisition coverage. The selected slice originated from a whole-body acquisition in 93.9% of cases (craniocaudal extent median 1,916 mm, IQR 1,776–2,024 mm), in which the geometric center reliably approximates the abdomen/pelvis; the remaining 6.1% were limited-coverage (“partial body”) acquisitions. MAE was stable across coverage: 4.02 kg for whole-body versus 4.67 kg for partial-body acquisitions, and 3.65–4.43 kg across tertiles of craniocaudal extent (**Table S3**). The absence of a precise anatomical landmark is examined further as a limitation in § 4.7.

##  Discussion

We developed and compared three approaches for automated body-weight prediction from a single middle-slice CT image: feature-engineered LightGBM, deep learning on images alone (ResNet-18 Images Only), and deep learning with late fusion of the same engineered features into the 512-dimensional image embedding (ResNet-18 CT Fusion). All models were selected by k-fold cross-validation on the combined train+validation development pool (30,857 slices) and evaluated, without further tuning, on a held-out test partition that was patient-disjoint from that pool. On the held-out test set, ResNet-18 CT Fusion achieved MAE 4.06 kg (95% CI 3.90–4.23; *R²* = 0.92), with 93.8% of predictions within ± 10 kg. A weighted ensemble of ResNet-18 CT Fusion and LightGBM (84/16) reduced MAE by 0.07 kg to 3.99 kg; the improvement was statistically significant by patient-level paired bootstrap (*p* < .001) but corresponds to approximately 0.1% of mean test-set body weight, below thresholds of practical relevance for the retrospective missing-weight imputation this method targets.

### Methodological contributions

The contribution of this study is methodological rather than a claim of superior predictive accuracy: where the full CT volume is available, a volumetric tabular-feature approach remains both simpler and more accurate (§ 4.3), and the value here is in characterising the single-slice regime and the role of metadata fusion within it. To our knowledge, this is the largest systematic comparison of gradient-boosting, deep-learning, and ensemble approaches for single-slice CT body-weight prediction reported to date (34,336 slices, 17,965 patients, a decade of routine PET/CT). Four methodological choices distinguish it: (i) all primary results are reported on a held-out test partition (*n* = 3,479, 1,796 patients) never used in training, model selection, hyperparameter tuning, or ensemble-weight optimization; the fixed validation partition, which is not independent of the fold-best models (§ 2.4.1), was used only during development; (ii) confidence intervals are computed at the patient level, handling the intra-patient correlation of multi-slice contributions (mean 1.9 examinations/patient); (iii) model comparison uses patient-level paired bootstrapping with two-sided p-values; and (iv) split-conformal prediction intervals, calibrated on out-of-fold residuals, provide per-case uncertainty with empirical test coverage rather than only group-level summaries.

### Accuracy versus precision

The mean test-set bias of − 0.09 kg for the CT Fusion + LightGBM ensemble (and the comparably small biases of the other models, all within ± 0.4 kg) reflects accuracy at the **population** level: the predictions are, on average, close to the true weights across the cohort. This is distinct from precision at the **individual case** level, which is more fully characterized by the 95% limits of agreement of approximately ± 11 kg and the split-conformal 95% prediction interval of width 19.8 kg with 93.8% test-set coverage (§ 3.5). Small population-level bias and wide individual-case bounds are not contradictory: the model is well-centered but the per-case dispersion is non-trivial. This distinction bounds the downstream utility of the predictions. To illustrate the magnitude, a 95% prediction interval of ± 9.9 kg on a mean weight of 77 kg corresponds to roughly ± 13% uncertainty in any quantity that scales linearly with body weight, which is too large for reliable per-case use. The predictions are therefore suited to population- and cohort-level applications, such as weight-stratified cohort analysis, where the small mean bias is the relevant quantity and per-case noise tends to cancel, rather than to individual-examination use.

### Comparison to prior work

Direct numerical comparison with prior CT-based weight-prediction studies is constrained because cohorts, input modalities, and evaluation designs differ substantially. The most directly relevant prior work is Ichikawa et al. [8], who used LightGBM on 11 volumetric tabular dose metrics extracted from whole-body PET/CT examinations to report MAE 1.99 kg on 3,996 Japanese patients. Our single-slice approach reports test-set MAE 4.06 kg for ResNet-18 CT Fusion on a substantially larger and demographically heterogeneous cohort (17,965 patients across a decade of acquisition). The two studies are not directly commensurable because Ichikawa et al. used whole-volume features and evaluated by cross-validation rather than held-out test, while our approach uses only a single axial slice and reports on a partition that was never used during training.

The accuracy gap can be partly decomposed. Our *own* LightGBM, applied to single-slice dose metrics, achieves MAE 5.61 kg, well above the same algorithm on Ichikawa’s whole-volume metrics (1.99 kg). Because the modeling approach is held constant, this 3.6 kg difference is attributable principally to the reduced information content of a single slice relative to a whole-volume feature set, rather than to the choice of model. This decomposition is indicative rather than exact: the two studies also differ in cohort size and demographic heterogeneity and in evaluation protocol (cross-validation versus held-out test), components that cannot be held constant across the comparison. Within the single-slice regime, adding the image to the tabular features recovers part of this gap (LightGBM 5.61 → ResNet-18 CT Fusion 4.06 kg, a 27.6% reduction), confirming that the image carries weight-predictive signal beyond the tabular metrics, but it does not close the gap to volumetric approaches. The practical implication is not a general efficiency advantage. A volumetric tabular-feature approach of the kind reported by Ichikawa et al. [8] uses no image-based deep learning and is both simpler and more accurate than the single-slice image models evaluated here, so where the full CT volume is available such an approach is preferable. Any efficiency argument for single-slice processing applies only within image-based deep-learning frameworks, where analysing one axial slice avoids the cost of volumetric or multi-slice convolutional inference. The chest-radiograph methods of [9] (MAE 2.63–3.35 kg) and scout-based approaches of [10] (MAE ~ 4–5 kg) similarly differ in cohort and design and should be compared with caution.

The dominance of water equivalent diameter (WED, 47.3% Gain importance) as the leading feature is consistent with AAPM Report 220 [2, 3], which establishes WED as the fundamental geometric metric for patient-size correction in CT dosimetry. The weight-prediction signal exploited in our models is essentially the same physical signal already used in routine SSDE calculation.

### Use case: retrospective data completeness

The motivating context is retrospective imaging archives in which body weight was not contemporaneously recorded. In such archives, the body-weight-dependent tasks noted in § 1.1 currently require either exclusion of examinations with missing weights or manual chart review at scale. A model that estimates body weight directly from the corresponding CT slice (MAE ~ 4 kg, 94% within ± 10 kg) substantially reduces both costs. The current study demonstrates the prediction methodology; validating the predictions within specific retrospective cohort analyses is left as future work.

Prospective weight-based applications (e.g., contrast-media volume selection and medication dosing in unconscious patients) are explicitly outside the scope of this study. Predictions should not be used in lieu of a scale measurement when one is available; the methods presented here address the case where scale measurement is missing or unrecoverable.

###  Model selection recommendation

For retrospective workflow integration we recommend ResNet-18 CT Fusion as the single-model primary choice. The CT Fusion + LightGBM ensemble’s 0.07 kg MAE improvement is reproducibly detectable but below practical relevance for the retrospective use cases described in § 4.4. The simpler single-model deployment removes the operational complexity of maintaining two independently trained models and their inference pipelines. The metadata-fusion benefit (14.2% MAE improvement over ResNet-18 Images Only on test) clearly justifies extracting the 19 CT-derived features, all of which are already available from routine DICOM headers and a single-slice analysis. Standalone LightGBM remains a reasonable choice in CPU-only or resource-constrained environments at the cost of moderately higher error (MAE 5.61 kg on test).

### Subgroup performance and practical implications

Subgroup analysis (§ 3.9, Table 4) showed strong performance in the 40–80 kg range and degradation at the weight extremes, where small or negative within-bin *R²* values (most notably − 0.539 at 100–120 kg) reflect within-bin weight variance comparable to the prediction noise: the model captures cross-cohort weight trends (test *R²* = 0.92, Table 2) but cannot finely rank-order patients within a narrow ~ 20 kg window. This motivates the case-level uncertainty flagging in § 4.7: predictions outside the 40–100 kg range should be treated as lower-confidence. Performance was equivalent across sexes and strong in the pediatric CV-pooled subgroup (the small test-partition pediatric sample limits the test-set claim); tube-voltage-stratified differences reflect the association between scanner-protocol selection and body habitus rather than a property of the model itself.

### Limitations

Several limitations constrain generalizability and warrant explicit acknowledgement.

(1) **Single-institution**,** single-modality cohort.** All data originate from one institution’s whole-body PET/CT archive. Multi-center validation across scanner manufacturers, patient populations, and imaging protocols is the essential next step.

(2) **Geometric middle-slice selection.** The selected slice is the geometric center of the scan volume rather than an anatomically standardized landmark (e.g., L3 vertebra). Different scan-coverage ranges yield slices at different anatomical levels, introducing variability that an L3-vertebra–based selection method would reduce. The current method was chosen for computational simplicity and reproducibility across diverse scan types; an L3-based extension is straightforward future work.

(3) **No domain-shift evaluation.** The model is not designed for amputees, severe edema, or other unusual body habitus, which were not separately characterized in the training cohort. Performance in such cases is expected to be poor and is not claimed.

(4) **Patient-level random partitioning without explicit stratification.** Train, validation, and test partitions are patient-disjoint and have comparable weight distributions (Table 1), but partitioning was random rather than explicitly stratified on weight, age, sex, or kVp. Under a deployment scenario with a markedly different weight distribution from the development cohort (e.g., a pediatric service or a bariatric-surgery service), performance is expected to differ from the test-set numbers reported here.

(5) **No external validation cohort.** Test-set numbers reported here describe **internal** generalization across patients within a single institution, not generalization across institutions or scanner generations. We have therefore avoided “external” or “out-of-distribution” language throughout.

(6) **Prediction-interval calibration.** Calibrating intervals naively on the fixed validation partition undercovers on test (90% PI 61.1%; 95% PI 75.5%), because the deployed k-fold-best models were exposed to most of that partition during fold training (§ 2.4.1). Calibrating split-conformal intervals on genuinely out-of-fold residuals instead restores close-to-nominal empirical test coverage (89.0% at the 90% level, 93.8% at the 95% level; Fig. S5). A small residual undercoverage remains, and the constant-width (marginal) form is conservative in the middle of the weight range; conditional-quantile or locally adaptive conformal intervals would be more efficient and are left to future work.

(7) **Architecture and hyperparameter search depth.** No formal architecture search (e.g., ResNet-34/50) or systematic hyperparameter optimization was performed; values were adopted from a brief fold-0 sweep and held fixed for fairness, and no formal sensitivity analysis over preprocessing choices or scanner-specific effects was conducted. A deeper-architecture ablation is left for future work.

### Future directions

The natural extensions of this work fall into three categories. First, multi-center external validation is the essential next step before the predictions are relied upon for the retrospective uses described in § 4.4. Second, validating the predictions in specific retrospective applications, such as weight-stratified cohort analysis and reconstruction of longitudinal weight trajectories across serial examinations, would establish their utility for concrete auditing tasks; the wide per-case interval means such uses are best suited to cohort-level trends or large weight changes rather than small individual differences. Third, conditional uncertainty quantification (e.g., quantile regression on residuals, Monte Carlo dropout, or evidential deep learning) would yield per-case prediction intervals adapted to local weight density, replacing the marginal (constant-width) conformal PIs reported here.

## Conclusion

We developed and compared three single-slice CT body-weight prediction methods (feature-engineered LightGBM, ResNet-18 on images alone, and ResNet-18 with late metadata fusion) on 34,336 examinations from 17,965 patients, with k-fold model selection on the combined train+validation development pool followed by unbiased evaluation on a patient-disjoint held-out test partition.

The methodologically important finding is the 14.2% MAE improvement on the held-out test set (Table 2) achieved by late fusion of 19 CT-derived tabular features into the ResNet-18 image embedding, demonstrating that scanner and acquisition metadata carry weight-predictive signal complementary to image features at minimal implementation cost. On the held-out test set, ResNet-18 CT Fusion achieved MAE 4.06 kg (95% CI 3.90–4.23; *R²* = 0.92) with 93.8% of predictions within ± 10 kg. A weighted ensemble with LightGBM reduced MAE by a further 0.07 kg with statistical significance but below practical relevance, supporting ResNet-18 CT Fusion alone as the recommended single-model solution.

The intended application is retrospective imaging archives with missing or unrecoverable body-weight records, where the method addresses a practical gap in data completeness for weight-complete cohort assembly, weight-stratified analysis, and AI dataset construction. Per-case uncertainty is quantifiable through out-of-fold split-conformal prediction intervals (90% PI ≈ ± 8 kg, 95% PI ≈ ± 10 kg), allowing predictions for atypical-body-habitus patients to be flagged for confirmatory measurement before downstream use.

Multi-center external validation, confirmation of utility in weight-stratified cohort analyses, and conditional uncertainty quantification are the natural next steps before the method is relied upon in practice.

## Supplementary Information

Below is the link to the electronic supplementary material.

Supplementary Material 1

## Data Availability

Patient imaging data are not publicly available due to institutional privacy requirements and ethical constraints on the de-identified retrospective clinical archive. De-identified aggregate metrics, model predictions, and tabular feature distributions supporting the findings reported in this manuscript are available from the corresponding author on reasonable request.

## Code availability

Analysis and training code, including model architectures, training scripts, and the held-out test-set evaluation pipeline, is available from the corresponding author on reasonable request.
