# Supplementary material for: Single-slice ct body-weight prediction with image and metadata fusion: a multi-model comparison
Source: Radiol Phys Technol. 2026 Aug 19;19(4):1588–601. doi: 10.1007/s12194-026-01122-1 (PMC13740028; doi:10.1007/s12194-026-01122-1)
Supplement: Supplementary file 1 [file 12194_2026_1122_MOESM1_ESM.docx]

# Supplementary Materials

Companion to: *Single-Slice CT Body-Weight Prediction with Image and Metadata Fusion: A Multi-Model Comparison*

This supplement contains supplementary figures (S1–S5) and supplementary tables (S1–S4) referenced in the main manuscript. Where indicated, supplementary figures use the 5-fold cross-validation out-of-fold pooled predictions (*n* = 30,857 from the combined train+validation development pool; see §2.4.1) rather than the held-out test partition (*n* = 3,479); this is to maintain per-subgroup statistical power for sparse strata (e.g., pediatric or low-weight subgroups). The held-out test partition is the primary, unbiased evaluation reported in the main text.

## Supplementary Figure S1. Per-fold ensemble weight optimization

***Fig. S1*** Per-fold ResNet-18 weight in the ResNet-18 CT Fusion + LightGBM ensemble. Weights were determined by 1% grid search on the validation partition of each fold (objective: validation MAE). Per-fold optimal ResNet-18 weights ranged 84–90% (mean 87%); per-fold MAE values are annotated above each bar. The narrow range demonstrates stable complementarity between the deep-learning and gradient-boosting models across folds. Referenced in main-text §2.3.4 (ensemble methods) and §3.4 (paired comparison)

## Supplementary Figure S2. ResNet-18 training curves

***Fig. S2*** Training and validation curves for the two ResNet-18 variants (Images Only, top row; CT Fusion, bottom row). Left column: MSE loss. Right column: MAE in kg. Both variants converged by approximately epoch 150 with no divergence between training and validation, confirming that dropout (*p* = .3), weight decay ($1\times{10}^{-4}$), and early stopping (patience 15) effectively prevented overfitting. The CT Fusion variant converged faster and to a lower validation loss than Images Only. Curves shown are from fold 3 (representative). Referenced in main-text §2.3.3 (training procedure)

## Supplementary Figure S3. Prediction error distributions

***Fig. S3*** Histograms of prediction error (predicted − actual, kg) for the four model families: LightGBM, ResNet-18 Images Only, ResNet-18 CT Fusion, and the optimized ensemble. All distributions are approximately normal and centered near zero, with the ensemble and ResNet-18 variants showing narrower distributions than LightGBM. A slight left skew (underestimation) is common to all models. Mean and SD are annotated in each panel legend. Companion to main-text **Fig. 1**, which presents the same distributions on the held-out test set

## Supplementary Figure S4. Absolute error statistics

***Fig. S4*** (a) Box plots of absolute prediction error by model on the 5-fold CV pooled predictions (*n* = 30,857). (b) Cumulative distribution functions of absolute error. The ensemble (green) achieves the highest cumulative proportion at every absolute-error threshold, with 73.0% of predictions within ±5 kg and 88.2% within ±10 kg. LightGBM (red) shows the longest tail. Companion to main-text **Fig. 2**, which presents the same analysis on the held-out test set

## Supplementary Table S1. Top 10 LightGBM feature importance values

***Table S1.*** Top 10 features ranked by Gain importance in the LightGBM model (5-fold mean). Water equivalent diameter (WED) and effective diameter (ED), both AAPM TG-220 patient-size metrics, together account for 63.7% of the model’s predictive signal, consistent with the size-specific dose theory behind the approach. Scanner generator power and body pixel count contribute additional morphometric signal. Referenced in main-text §3.8 (feature importance).

| Rank | Feature | Importance (Gain %) |
| --- | --- | --- |
| 1 | Water Equivalent Diameter (WED) | 47.3 |
| 2 | Effective Diameter (ED) | 16.4 |
| 3 | Generator Power (kW) | 7.9 |
| 4 | Number of Body Pixels | 6.5 |
| 5 | Table Height | 3.6 |
| 6 | X-ray Tube Current | 2.5 |
| 7 | Exposure (mAs) | 1.9 |
| 8 | Tube voltage (kVp) | 1.7 |
| 9 | CTDIvol | 1.7 |
| 10 | Total Tissue Area | 1.6 |

## Supplementary Figure S5. Prediction-interval reliability

***Fig. S5*** Reliability diagram for ResNet-18 CT Fusion prediction intervals on the held-out test set: nominal coverage (x-axis) versus empirical test-set coverage (y-axis). Split-conformal intervals calibrated on out-of-fold residuals (green) track the identity line closely (90% → 89.0%, 95% → 93.8%), whereas intervals calibrated naively on the fixed validation partition (red) substantially undercover (90% → 61.1%, 95% → 75.5%) because the deployed fold-best model was exposed to most of that partition during fold training. Referenced in main-text §2.5, §3.5, and §4.7.

## Supplementary Table S2. One-examination-per-patient sensitivity analysis

***Table S2.*** Held-out test performance with the test partition restricted to a single examination per patient (earliest study date; *n* = 1,796), compared with the full test partition (*n* = 3,479). All models change by ≤0.08 kg in MAE and show marginally higher *R²*, confirming repeated examinations do not inflate the reported performance. Referenced in main-text §3.11 (R1.M2).

| Model | MAE full (kg) | MAE 1/patient (kg) | *R²* full | *R²* 1/patient | ±5 kg full | ±5 kg 1/patient |
| --- | --- | --- | --- | --- | --- | --- |
| LightGBM | 5.61 | 5.59 | .856 | .859 | 56.5% | 56.2% |
| ResNet-18 Images Only | 4.73 | 4.65 | .893 | .897 | 63.3% | 64.5% |
| ResNet-18 CT Fusion | 4.06 | 4.02 | .922 | .925 | 69.5% | 70.0% |
| Ensemble (Images Only + LightGBM, 66/34) | 4.33 | 4.27 | .908 | .912 | 67.8% | 68.5% |
| Ensemble (CT Fusion + LightGBM, 84/16) | 3.99 | 3.95 | .924 | .927 | 70.7% | 71.4% |

## Supplementary Table S3. Held-out test performance by scan coverage

***Table S3.*** ResNet-18 CT Fusion and ensemble MAE on the held-out test set stratified by acquisition coverage. Performance is stable across coverage strata, supporting the geometric middle-slice choice for the predominantly whole-body cohort while quantifying the modestly higher error for limited-coverage acquisitions. Craniocaudal extent = number of slices in series × slice thickness. Referenced in main-text §2.2.2 and §3.11 (R1.M1, R3.2).

| Subgroup | *n* | CT Fusion MAE (kg) | CT Fusion ±5 kg | Ensemble MAE (kg) |
| --- | --- | --- | --- | --- |
| Whole-body (body part = ABDOMEN) | 3,267 | 4.02 | 69.8% | 3.95 |
| Partial-body acquisition | 212 | 4.67 | 64.6% | 4.65 |
| Craniocaudal extent: short (4–1,840 mm) | 1,185 | 4.11 | 69.5% | 4.01 |
| Craniocaudal extent: medium (1,848–1,952 mm) | 1,144 | 3.65 | 73.6% | 3.48 |
| Craniocaudal extent: long (1,960–3,932 mm) | 1,150 | 4.43 | 65.4% | 4.48 |

## Supplementary Table S4. Cross-validation-selected vs full-pool retrained models

***Table S4.*** Held-out test performance of the deployed cross-validation-selected (k-fold-best) models versus the same architectures retrained on the full training partition (27,329 slices) after model selection, evaluated on the identical held-out test partition (*n* = 3,479). The two selection strategies give comparable test performance, confirming the fold-selection rule is not a source of optimistic bias. Referenced in main-text §2.4.1 (R1 Minor 1).

| Model | Selection strategy | Test MAE (kg) | Test RMSE (kg) | Test *R²* |
| --- | --- | --- | --- | --- |
| ResNet-18 CT Fusion | k-fold-best (fold 3) | 4.06 | 5.65 | .92 |
| ResNet-18 CT Fusion | retrained on full training partition | 4.13 | 5.70 | .92 |
| ResNet-18 Images Only | k-fold-best (fold 0) | 4.73 | 6.62 | .89 |
| ResNet-18 Images Only | retrained on full training partition | 4.47 | 6.26 | .90 |
